# Exon creation and establishment in human genes

**DOI:** 10.1186/gb-2008-9-9-r141

**Published:** 2008-09-23

**Authors:** André Corvelo, Eduardo Eyras

**Affiliations:** 1 Computational Genomics, Universitat Pompeu Fabra, Dr. Aiguader 88, Barcelona, 08003, Spain; 2 Graduate Program in Areas of Basic and Applied Biology, Universidade do Porto, Praça Gomes Teixeira, Porto, 4099-002, Portugal; 3 Catalan Institution for Research and Advanced Studies, Passeig Lluís Companys 23, Barcelona, 08010, Spain

## Abstract

**Background:**

A large proportion of species-specific exons are alternatively spliced. In primates, *Alu *elements play a crucial role in the process of exon creation but many new exons have appeared through other mechanisms. Despite many recent studies, it is still unclear which are the splicing regulatory requirements for *de novo *exonization and how splicing regulation changes throughout an exon's lifespan.

**Results:**

Using comparative genomics, we have defined sets of exons with different evolutionary ages. Younger exons have weaker splice-sites and lower absolute values for the relative abundance of putative splicing regulators between exonic and adjacent intronic regions, indicating a less consolidated splicing regulation. This relative abundance is shown to increase with exon age, leading to higher exon inclusion. We show that this local difference in the density of regulators might be of biological significance, as it outperforms other measures in real exon versus pseudo-exon classification. We apply this new measure to the specific case of the exonization of anti-sense *Alu *elements and show that they are characterized by a general lack of exonic splicing silencers.

**Conclusions:**

Our results suggest that specific sequence environments are required for exonization and that these can change with time. We propose a model of exon creation and establishment in human genes, in which splicing decisions depend on the relative local abundance of regulatory motifs. Using this model, we provide further explanation as to why *Alu *elements serve as a major substrate for exon creation in primates. Finally, we discuss the benefits of integrating such information in gene prediction.

## Abstract 2

A comparative genomics study of alternatively spliced exons showing that the relative local abundance of splicing regulatory motifs influences splicing decisions in humans.

## Background

It is well established that alternative splicing (AS) is a widespread mechanism responsible for increased protein diversity and complexity among eukaryotes. The importance of this mechanism in the regulation of gene function has raised the question of its role in the context of evolution. Recent studies separating exons by evolutionary ages have shown that species-specific exons are mostly alternatively spliced [1,2] and previous analyses have shown that the converse seems to be the case, that is, many alternative exons are species-specific [3,4]. Moreover, evolutionary rate measurements show differences between alternatively and constitutively spliced regions [5,6]. These have been linked to positive selection on alternatively spliced regions that accelerates the evolution of protein sequences [7,8] and to a selective constraint due to splicing regulation [9-11]. Thus, changes in the content of splicing regulatory motifs play an important role in shaping the exon-intron structures of genes. In particular, these changes give rise to species-specific exons, which can account for phenotypic variations between organisms [12]. These exons may occur as fortuitous additions to existing transcripts, but they confer an opportunity to explore new functions with negligible disruption of the usual protein function [3]. The study of the mechanisms by which these species-specific exons can appear and become established is therefore key for the understanding of splicing regulation.

Three main mechanisms have been identified as being responsible for the appearance of new exons: gene duplication events, tandem exon duplication events [13], and exaptation, whereby a genomic sequence that did not function as an exon becomes exonized. This last mechanism is mostly driven by transposable elements (TEs) in mammals [14-18]. In particular, *Alu *elements play a prominent role in exon creation in primates [19-21]. These elements have motifs that resemble splice sites as part of their consensus sequence, especially in the opposite orientation, which can become functional through specific mutations [22-24], allowing exonization of part of the element. RNA editing has also been identified as a mechanism triggering exon creation from *Alu *elements [25]. In this case, however, the splice site is not in the genomic sequence, but it is instead created during the RNA editing process.

The fact that species-specific exons are, in general, poorly included suggests that they mainly appear with weakly recognized splicing signals. In particular, this is the case for some examples of exonized *Alu *elements [20], for which the strength of the base pairing between the U1 snRNA and the functional 5' splice site of the *Alu *determines the level of inclusion [23]. Although alternative exons are generally associated with weaker splice sites compared to constitutive exons [26], the distributions of splice site scores for both types of exon greatly overlap, suggesting that the strength of the splice site alone cannot explain the observed differences in inclusion levels between species-specific and evolutionarily conserved exons. Indeed, splice sites are not the only signals governing the recognition of an exon. There are also splicing enhancers and silencers, which function as activators and repressors of the splicing mechanism, respectively. These can occur in exons as exonic splicing enhancers (ESEs) or silencers (ESSs), and in introns as intronic splicing enhancers or silencers. Many of these regulators have been identified using experimental [27] and computational [28,29] methods, and recent analyses have recognized their changing role depending on their position along the exon or the intron [30,31]. These results highlight the variety of sequences that can function as splicing *cis*-regulatory elements, and their position-specific effects. This raises the question of whether the low inclusion observed for species-specific exons is related to a form of splicing regulation that is essentially different from that of evolutionarily conserved exons. Moreover, it is known that for alternative exons the density of ESEs is significantly lower compared with constitutive ones [29,32]. However, the minimal splicing regulatory requirements for *de novo *exonization are poorly understood and it is not yet known how this regulation changes with exon age.

In this article we investigate the regulatory content governing the definition of the new exon and how the splicing regulatory properties of exons change with time. Additionally, we show how the local differences in the density of splicing regulatory motifs characterize real exons with respect to pseudo-exons better than taking into account the exonic or intronic content alone. Finally, we study the case of *Alu *exonization, complementing prior analyses [33-36], and provide further explanations as to why this element is the most commonly exonized.

## Results

### Three age sets

We separated a set of internal and fully protein-coding human exons into three age groups according to their presence or absence in other species. We classified exons as primate specific (PS) if they were found in human but not in mouse and cow; mammalian specific (MS) if they were found in human, mouse and cow, but not in chicken or Tetraodon; and vertebrate and older (VO) if they were found in all these five species. Using this approach (see Materials and methods for details) we collected three mutually exclusive sets of 359 PS exons, 323 MS exons and 13,249 VO exons. Additionally, we did not include any exons for which the expressed sequence tag (EST) or cDNA evidence indicated variable splice sites. These sets represent human protein-coding exons of three different ages and constitute the basis of our analysis.

**Figure 1:**
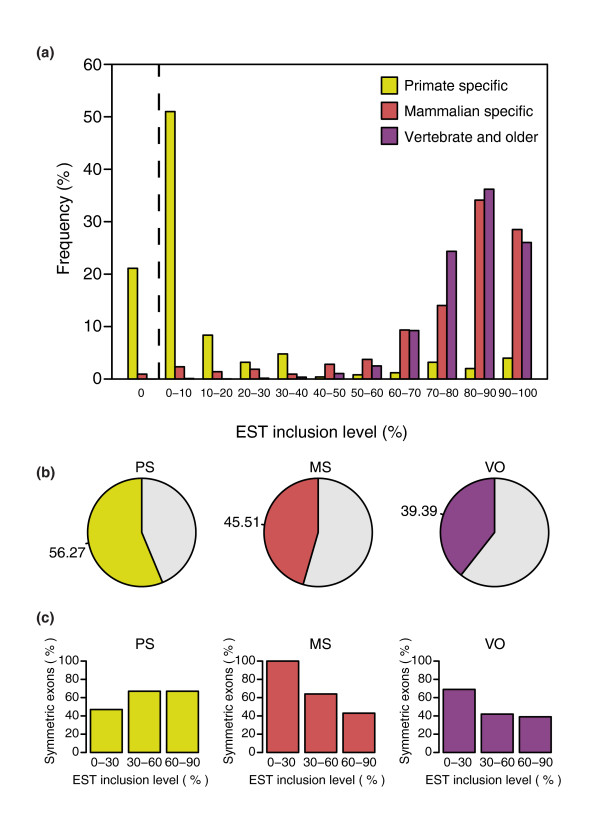
EST inclusion level and symmetry. **(a) **EST inclusion levels for the three age groups. The x-axis shows the inclusion levels in ranges of 10, and the y-axis shows the proportion of exons from each subset falling within each range. For each exon, the EST inclusion level is defined as N_i_/(N_i _+ N_s_) × 100%, where N_i _is the number of ESTs including the exon and N_s _the number of ESTs skipping the exon. Only exons with N_i _+ N_s _≥ 10 were considered. On the left of the dashed line we plot the frequencies for exons with zero EST inclusion level. **(b) **Percentage of symmetric exons (length multiple of three) for each age group. **(c) **Percentage of symmetric exons by EST inclusion level category for each age group. Only alternative spliced exons with N_i _+ N_s _≥ 10 were considered.

For the three categories, we calculated inclusion levels using ESTs. In accordance with previously published results for PS cassette exons [1,2], our PS exons have lower EST inclusion levels than MS and VO exons (Mann-Whitney, *p *= 7 × 10^-65^), whereas these two other sets show no significant differences (Figure 1a). PS exons are included, on average, in less than 10% of the transcripts, with only about 5% of them being constitutive. Even though PS exons are included at very low frequencies, the pressure for reading frame maintenance is higher than in MS and VO exons (Chi-square, *p *= 0.006 and *p *= 1.6 × 10^-10^, respectively; Figure 1b). More than half of PS exons (56.27%) have a length multiple of three, also called symmetric. On the other hand, the percentages of MS (45.51%) and VO (39.39%) symmetric exons are smaller. It has been previously reported that conserved alternative exons present a bias towards symmetry [6,37,38]. As most of the PS exons are alternative, these numbers could just reflect a relationship between reading frame preservation and inclusion levels, regardless of exon age. We thus investigated the relationship between exon symmetry and EST inclusion levels for alternative exons belonging to the three age groups. MS and VO exons tend to be more frequently symmetric at lower inclusion levels (Chi-square, *p *= 1.7 × 10^-4 ^and *p *= 3.4 × 10^-3^, respectively; Figure 1c). This agrees with previous reports of a bias towards symmetry in evolutionarily conserved alternative exons [37,38]. However, we observed the opposite behavior for PS exons, although the observed differences are not significant, probably due to the small number of cases in the high inclusion level categories. This suggests that the pressure for reading frame maintenance may be related to exon age. A study of the dependency on the inclusion level would require further analysis with larger sets of exons.

### Exon creation from repetitive sequences

**Figure 2:**
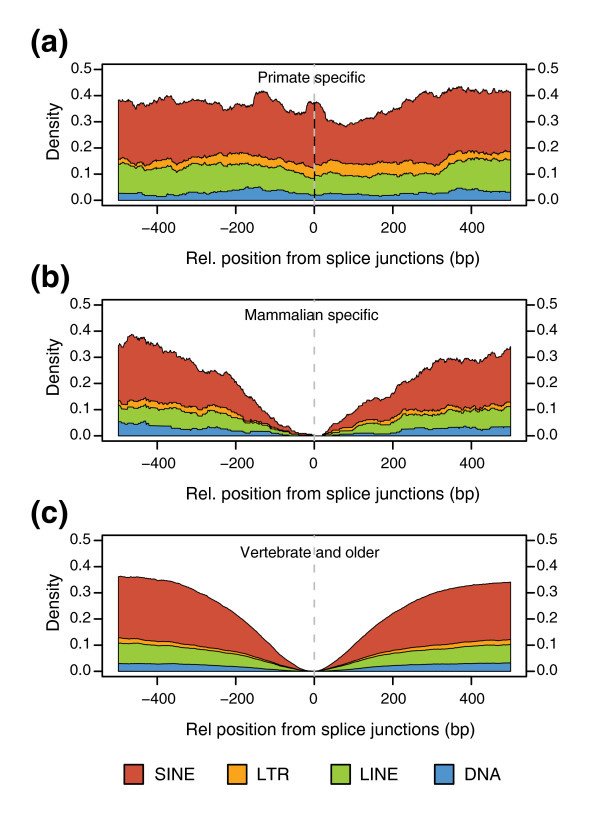
Intronic densities for the main classes of repetitive elements. **(a) **Primate specific, **(b) **mammalian specific and **(c) **vertebrate and older. At each intronic position, the density was calculated as the proportion of cases in which the base was covered by a given type of repetitive element. We give on the x-axis the relative position from the splice junctions as negative if upstream of the acceptor site or positive if downstream of the donor site.

Along with tandem duplication events [13], exonization of TEs is one of the most important mechanisms of exon creation [17,35,39,40]. Therefore, we assessed the overlap between exons from the three age sets and TEs, considering as overlap the cases in which the TE covers at least one of the splice sites. We found that PS exons have a high density of TEs in their flanking intronic regions (Figure 2a) and about 43% of the cases overlap TEs (Table 1). On the other hand, MS and VO exons have a very low density of TEs in the proximal adjacent intronic regions (Figure 2b, c) and show negligible overlap of TEs with their splice sites. Additionally, excluding the eight cases in which the exon overlaps more than one TE, we found that for 116 (79.5%) of the PS exons overlapping TEs, the TE is in the opposite strand of the exon. Although *Alu *elements, unlike other TEs such as L1 and Long Terminal Repeat (LTR) retrotransposons [41], were not found to have a bias in the strand of insertion in human introns [40], we find that most of the *Alu *elements (88.3%) overlapping a PS exon occur in the strand opposite to the gene (anti-sense). In only 9 out of the 77 cases (11.7%) we found sense *Alu *elements, and in only 4 of these is the overlap complete. Moreover, the percentage of anti-sense cases for non-*Alu *TEs is 69.6%. This suggests that for TEs and, especially, for *Alu *elements, although insertion can potentially occur in either strand, exonization occurs mainly in the opposite strand. Interestingly, although we found no overlap in the MS set, we found 19 cases (less than 0.15%) in the VO set; many of these were simple-repeats (Table 1). More details about the type of TE overlap are given in Table A1 in Additional data file 1. Remarkably, more than 50% of PS exons do not overlap a TE and cannot be explained by tandem duplication, as those cases were discarded during the exon classification.

**Table 1:** Overlap with repetitive elements

		SINE							
								
Exon set	N	*Alu*	Other	LINE	DNA	LTR	Other	Mixed	Total
PS	359	77	17	27	10	15	-	8	154
		21.45%	4.74%	7.52%	2.79%	4.18%		2.23%	42.90%
MS	323	-	-	-	-	-	-	-	
VO	13,249	-	5	14	-	-	10	-	29
			0.04%	0.11%			0.08%		0.22%

For each age group, we give the number and corresponding percentage of exons that overlap with different repetitive elements: SINEs (*Alu *and other), LINE, DNA, LTR and Other. Eight PS exons overlap more than one element (Mixed). We count as overlap when the element covers at least one of the splice sites of the exon.

### Analysis of the splicing regulatory content of exons

In order to understand the properties of the splicing regulatory content that determine the observed differences in inclusion between exon sets, we conducted an analysis of splicing *cis*-regulatory elements in exons and their flanking introns. For this analysis we used three sets of splicing regulatory elements (SREs): 666 ESE hexamers [42], which we call ESEcomb; all possible words obtained from the four position-specific weight matrices for SR-protein binding sites from ESE-finder (SF2/ASF, SC35, Srp40 and Srp55) using the proposed thresholds [43], which we call SRall; and 386 ESS hexamers [42], which we call ESScomb (see Materials and methods for a detailed description). Previous research has pointed out that ESEs are generally more abundant in exons than in introns [29,32,44], whereas ESSs are generally more frequent in introns than in exons [29,31]. In fact, some of the sets used here were partially defined based on exon/intron and on exon/pseudo-exon enrichment [28,29]. In order to better understand how these motifs distribute on both real/pseudo-exons and introns, we defined a set of real exons making use of the total set of exons from the three age groups. Additionally, we built a set of pseudo-exons from intronic regions that fall between protein-coding exons and are devoid of TEs (pseudo-INT). For both real and pseudo-exons, density profiles for each SRE set are plotted in Figure A1 in Additional data file 1. Real exons, as expected, show higher ESEcomb exonic densities when compared to pseudo-exons. Interestingly, the densities are lower in adjacent intronic regions. The inverse seems to be true for ESScomb. Relative to SRall, only intronic differences were observed between real and pseudo-exons.

This pattern suggests that the previously reported differences between exonic and intronic content in real exons, something not observed in pseudo-exons, are not merely due to an increase of ESEs and a decrease of ESSs in the exonic regions, but also to opposite changes in the adjacent intronic regions. Taking this into account, it is plausible to hypothesize that the effect exerted by SREs is context dependent. Splicing decisions depend on the correct discrimination between exonic and intronic regions and this is ultimately determined by sequence features and their positioning relative to the splice sites. Therefore, we define a measure, the exonic relative abundance (ERA), which encapsulates both exonic and intronic information. This measure is defined for each exon as the relative difference between exonic and intronic densities for a given set of regulators (see Materials and methods for details). This measure is such that, for signals that are more abundant in the exon than in the flanking intronic region, it takes on positive values. On the other hand, for signals that are more abundant in the flanking introns, the ERA values distribute around a negative mean. In addition, and contrary to the overall exonic or intronic density, this measure does not depend on SRE set size, which makes it useful for comparing the contribution from different SRE sets to the splicing phenotype.

### Relative abundance of splicing regulators improves the discrimination between real and pseudo-exons

**Figure 3:**
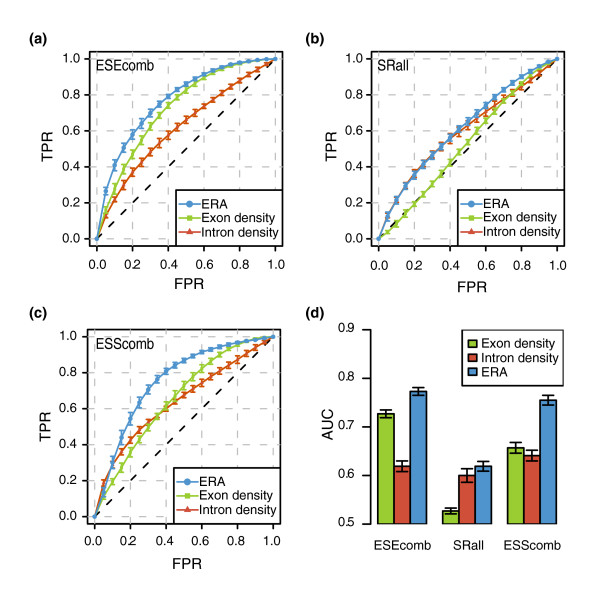
Performance comparison in real/pseudo-exon discrimination between different measures. ROC curves (vertically averaged) for exonic density, intronic density and ERA, using **(a) **ESEcomb, **(b) **SRall and **(c) **ESScomb as informative features. The average was calculated from 10 different subsets of the data (see text for details). **(d) **The corresponding AUCs. The error bars represent the standard error. FPR, false positive rate; TPR, true positive rate.

We find that the ERA can discriminate better between real and pseudo-exons than the overall density measures. For this analysis, we considered 10,000 real exons sampled from our three age groups and 10,000 pseudo-exons sampled from the pseudo-INT set. Each set was randomly split into 10 non-redundant groups. For each SRE set (ESEcomb, SRall and ESScomb), we scored the exons on three measures: exonic density; intronic density; and ERA. Figure 3 shows the receiver operating characteristic (ROC) curves for each of the SRE sets (Figure 3a–c), vertically averaged on each false positive rate (FPR) for the 10 subsets, and the corresponding areas under the curve (AUCs) (Figure 3d). These ROC curves allow comparison between classifiers for all possible thresholds and AUCs summarize global performance. We also used the 10 splits for a 10-fold cross-validation test; for each group used as a test set we used the other 9 as training sets. Accuracy results and corresponding thresholds of the tests can be found in Table 2 (see Table A2 in Additional data file 1 for the complete list of accuracy values using combined and individual SRE sets). The precision-recall curves for each classifier can be found in Figure A2 in Additional data file 1.

**Table 2:** Mean thresholds and accuracy for pseudo/real exon classification (10-fold cross-validation)

		Threshold		Accuracy	
			
SRE set	Measure	Mean	SD	Mean	SD
ESEcomb	Exonic density	0.564*	0.000	0.672	0.008
	Intronic density	0.450^†^	0.010	0.588	0.010
	Exonic relative abundance	0.136*	0.014	0.699	0.009
					
SRall	Exonic density	0.486*	0.003	0.535	0.006
	Intronic density	0.481^†^	0.002	0.585	0.010
	Exonic relative abundance	0.163*	0.019	0.580	0.012
					
ESScomb	Exonic density	0.358^†^	0.017	0.613	0.013
	Intronic density	0.492*	0.005	0.614	0.009
	Exonic relative abundance	-0.216^†^	0.007	0.707	0.010

*Minimum score cut-off for predicted real exons. ^†^Maximum score cut-off for predicted real exons.

We observe that ESEcomb exonic density performs, in general, better than intronic density (AUC, 0.727 and 0.619, respectively; Figure 3a). Surprisingly, we found that the opposite occurs for SRall at almost all FPR values (Figure 3b). That is, the intronic density of SRall is more informative than the exonic densities. Regarding ESScomb, even though exonic and intronic densities show different behaviors (Figure 3c), no differences in AUCs were observed. Interestingly, we found that ESEcomb and ESScomb perform better than each individual set from which they were built and consistently better than SRall (see Table A2 in Additional data file 1 for the performances of the individual sets).

Moreover, we found that ERA performs superiorly in discriminating real from pseudo-exons than intronic and exonic densities independently, on both ESEcomb and ESScomb sets at all FPR values (AUC, 0.773 and 0.755). Additionally, ERA (AUC, 0.619) provides a marginal improvement with respect to the information provided by the intronic density of SRall (AUC, 0.600).

### Differences in the relative abundance of regulators with age and exon establishment

In order to investigate the regulatory features that determine the observed differences in EST inclusion levels between recently created and older exons, we studied the splice site strengths for each exon group. The distributions of the splice site score for the three age groups, calculated as the sum of the acceptor and donor scores for each exon, can be found in Figure A3A in Additional data file 1. PS exons show significantly weaker splice sites (mean = 5.061; Mann-Whitney, *p *= 1.18 × 10^-8^) than MS (6.907) and VO (7.394) exons. Moreover, the difference between the MS and VO groups was also found to be significant (Mann-Whitney, *p *= 3.63 × 10^-3^). These differences are mainly supported by lower frequencies of pyrimidines upstream of the acceptor site and also by more degenerated donor signals in PS exons (Figure A3B in Additional data file 1). This suggests that the observed differences in exon inclusion may be related to the differences in splice site strength. However, these distributions largely overlap. We also observe that EST inclusion levels for PS exons seem to be more dependent on the splice site score than for MS or VO exons. Still, no clear, strong correlation between these two variables could be observed (Spearman's rank correlation, PS rho = 0.22, *p *= 3.81 × 10^-5^; MS rho = 0.12, *p *= 0.026; and VO rho = 0.09, *p *= 2.23 × 10^-27^). Thus, the change from low to high inclusion cannot be fully attributed to an increase in splice site strength.

**Figure 4:**
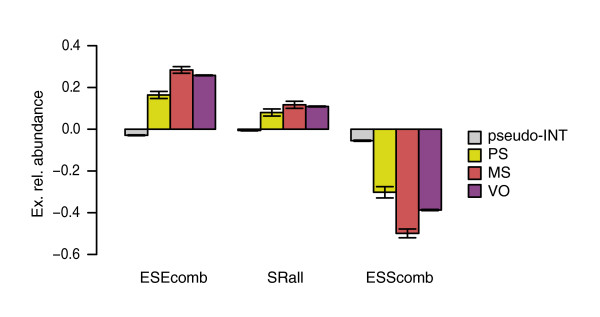
SRE ERA changes with age. Mean exonic relative abundance values for the three age groups (PS, MS and VO) and a set of pseudo-exons not overlapping any repeats (pseudo-INT) calculated for the three motif sets (ESEcomb, ESScomb and SRall). Exons overlapping *Alu *elements were excluded from the PS set. The standard error is also shown.

Accordingly, we considered SREs as additional contributors to the splicing phenotype. We calculated ERA values for each age group of exons (Figure 4), for the same SRE sets as before. As a control, we used the set of pseudo-exons not overlapping TEs, which we determined before (pseudo-INT). Figure 4 shows that pseudo-exons have ERA values distributed around zero for all SREs tested (ESEcomb, -0.029; SRall, -0.006; and ESScomb, -0.055). On the other hand, all real exons show positive values for ESEs and negative for ESSs. In particular, PS exons show the closest ERA values to pseudo-INT, but they are still significantly different (Mann-Whitney, ESEcomb *p *= 1.45 × 10^-20^, SRall *p *= 2.88 × 10^-8^, and ESScomb *p *≈ 0). Interestingly, we also observe differences between PS exons and MS/VO for two out the three SRE sets used. For ESEcomb and ESScomb, PS exons show lower absolute ERA values (0.164 and -0.302, respectively) than MS (0.284 and -0.499) and VO (0.258 and -0.387) (see Table A3 in Additional data file 1). Relative to SRall, no significant differences between age groups were observed. ERA was also calculated for the individual SRE sets (see Materials and methods for details). These results can be found in Figure A4 in Additional data file 1.

Focusing on MS and VO exons, we observe a surprising difference in the content of ESScomb motifs. VO exons present lower absolute ERA values than MS (Mann-Whitney, *p *= 3.06 × 10^-10^). This result derives from the fact that VO exons show relatively higher exonic densities of ESSs (0.272) compared to MS (0.213), while for intronic content no significant differences were found (Table A3 in Additional data file 1). Also, VO exons show slightly lower exonic densities for ESEcomb with respect to MS (MS 0.665, and VO 0.633; Mann-Whitney, *p *= 4.56 × 10^-6^). These results can be partially explained by the fact that VO exons have stronger splice sites. On the other hand, it also suggests that AS of VO exons may be more dependent on ESS content.

**Figure 5:**
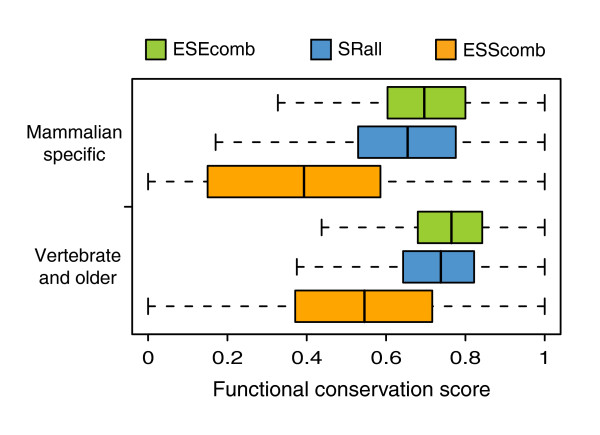
SRE functional conservation between human and mouse. SRE FCS between human and mouse of exonic regions covered by ESEcomb, SRall and ESScomb motifs for mammalian specific and vertebrate or older exons. See Materials and methods section for formula.

In order to understand if these regulatory elements were under different, possibly functional, constraints depending on the exon age, we investigated their conservation in the mouse orthologous exons (Figure 5). For this purpose, we have calculated the functional conservation score (FCS; see Materials and methods for detailed description) for all three SRE sets on both MS and VO exon sets. This measure reflects the fraction of nucleotides that are covered by motifs from the same SRE set in both human and mouse. This measure correlates with the percentage of sequence conservation but also takes into account cases where a substitution does not change the regulatory character of a region. In general, VO exons have higher FCS values compared to MS exons for ESEcomb (Mann-Whitney *p *= 8.42 × 10^-13^), SRall (*p *= 4.64 × 10^-14^) and ESScomb (*p *= 2.99 × 10^-16^). Additionally, FCS is higher for ESEcomb than for ESScomb for both MS and VO exons (Mann-Whitney, *p *≈ 0), which might reflect the importance of the conservation of the amount and position of ESEs in exons. In summary, although VO exons have lower density of ESEs, these are more conserved than in MS exons, indicating that ESE turnover is more frequent in MS compared to VO exons, in agreement with recent analyses [45]. Moreover, VO exons present a larger fraction of ESSs that are highly conserved, suggesting possible constraints due to AS regulation.

**Figure 6:**
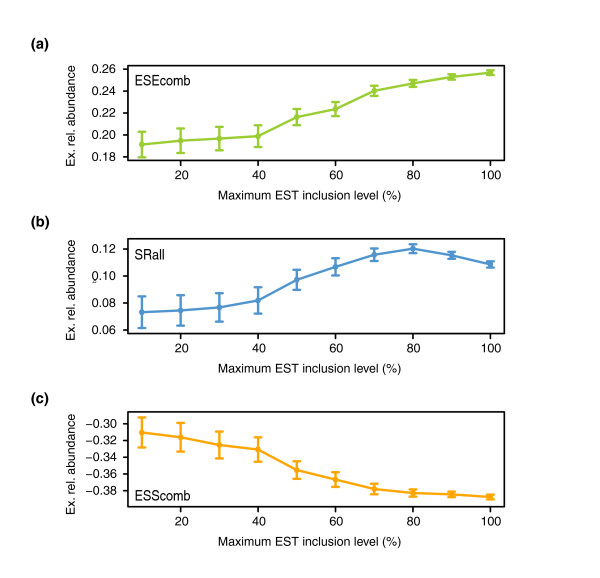
SRE exonic relative abundance and EST inclusion levels. Cumulative plot of ERA variation (y-axis) for bins of increasing maximum EST inclusion levels (x-axis) for **(a) **ESEcomb, **(b) **SRall and **(c) **ESScomb. The standard errors are also shown.

Interestingly, considering all exons from the three age groups, ERA values tend to increase for ESEs (ESEcomb and SRall) and decrease for ESSs (ESScomb). Figure 6 shows the mean ERA values plotted for bins of increasing EST inclusion levels. For ESEcomb (Figure 6a) and SRall (Figure 6b) we observe a consistent increase except at high EST inclusion levels, where SRall values slightly decrease. On the other hand, there is a consistent decrease for ESScomb at all EST inclusion levels (Figure 6c). Exonic and intronic densities do not show such gradients with EST inclusion levels (data not shown). Thus, inclusion levels seem to be determined by the local differences in the densities of motifs.

### Study case: why *Alu *elements are a good substrate for exonization

It has been recently reported that all TEs have approximately the same exonization levels with the exception of *Alu *elements, which are almost three times higher than other TE families [40]. Additionally, the high number of *Alu *copies in the human genome and their propensity to accumulate in intronic regions[40] make this element the main source of new exons originating from TEs. It has been shown that in some cases, cryptic splice sites are enough to incorporate part of an *Alu *element in the mature transcript [22,23] and that in other cases, specific splicing enhancers are needed for their inclusion [34]. We thus applied the ERA measure in order to understand which regulatory features, besides the presence of splice sites, may be responsible for the increased *Alu *exonization rate.

**Figure 7:**
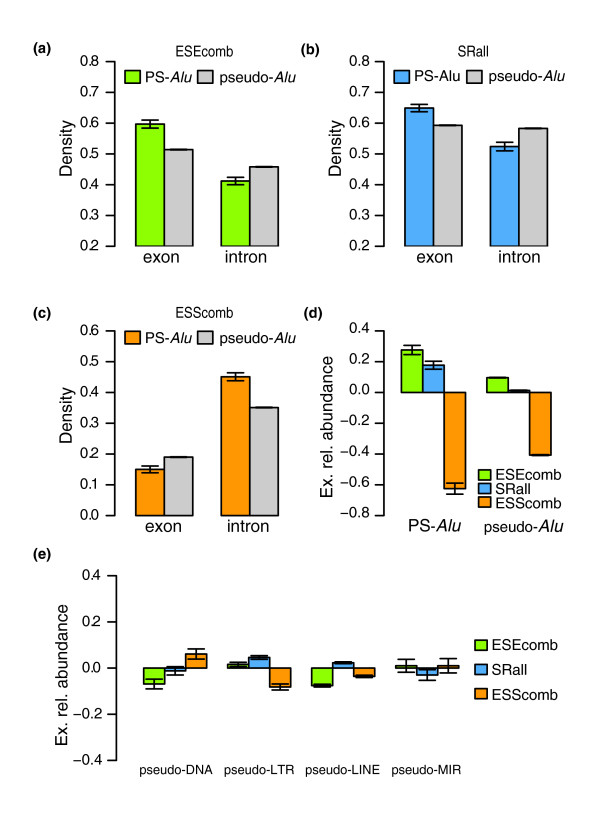
*Alu*'s unique *cis*-regulatory context. Exonic and intronic densities of **(a) **ESEcomb, **(b) **SRall and **(c) **ESScomb motifs on primate specific exons overlapping *Alu *elements (PS-*Alu*) and on *Alu *pseudo-exons (pseudo-*Alu*). **(d) **Exonic relative abundance of ESEcomb, SRall and ESScomb motifs for primate specific exons overlapping *Alu *elements (PS-*Alu*) and for *Alu *pseudo-exons (pseudo-*Alu*). **(e) **Exonic relative abundance for the same sets of motifs in pseudo-exons overlapping other classes of repeats, namely DNA, LTR, LINE and SINE non-*Alu *(MIR) repeats. The error bars represent the standard error.

We compared the SRE densities between the subset of PS overlapped by *Alu *elements (PS-*Alu*) and a set of *Alu *pseudo-exons bigger than 80 bp (pseudo-*Alu*) (see Materials and methods for details). Figure 7a, b show the mean exonic and intronic densities of the two ESE sets considered (ESEcomb and SRall) for PS-*Alu *and pseudo-*Alu*. The mean exonic densities of ESEcomb and SRall for PS-*Alu *(0.597 and 0.649, respectively) were significantly higher (Mann-Whitney, *p *= 4.89 × 10^-12 ^and *p *= 9.78 × 10^-6^) than the mean exonic densities for pseudo-*Alu *(0.514 and 0.593). Relative to ESScomb (Figure 7c), PS-*Alu *shows a mean value of exonic density of 0.150 while pseudo-*Alu *shows a mean value of 0.190 (Mann-Whitney, *p *= 1.09 × 10^-4^).

Surprisingly, we observe the opposite behavior when considering adjacent intronic regions. The mean values of the intronic density of ESEs are significantly lower for PS-*Alu *when compared to pseudo-*Alu *(Mann-Whitney, ESEcomb *p *= 3.64 × 10^-4 ^and SRall *p *= 2.02 × 10^-5^), while for ESScomb the mean density values are higher (Mann-Whitney, *p *= 1.12 × 10^-11^). All these results suggest that ESEs and ESSs play a role in *Alu *exonization. In Figure 7d we can observe that for PS-*Alu*, the mean ERA values for ESEcomb and SRall distribute around positive values (0.276 and 0.177) while the ESScomb values tend to distribute around a negative mean (-0.625). The absolute values are significantly greater than those obtained for pseudo-*Alu *(Mann-Whitney, *p *= 8.26 × 10^-10^, *p *= 1.31 × 10^-7 ^and *p *= 3.75 × 10^-10^). Furthermore, the fact that ESScomb produces the greatest difference of means suggests that this sequence feature might be the main determinant in the exonization of *Alu *elements. Comparing PS exons overlapped and non-overlapped by *Alu*s, we observe that the latter have higher exonic (0.247) and lower intronic (0.383) densities for ESScomb (Mann-Whitney, *p *= 6.29 × 10^-8 ^and *p *= 1.83 × 10^-4^, respectively). Consequently, their absolute ERA mean values (-0.302) are lower than those observed for *Alu *overlapped exons and, surprisingly, lower than those observed for pseudo-*Alu *(-0.407) (Mann-Whitney, *p *= 3.94 × 10^-10 ^and *p *= 6.03 × 10^-5^).

Finally, in order to test whether the found properties are *Alu *specific, we analyzed sets of pseudo-exons overlapping the other major families of mobile elements in the human genome: Long Interspersed Nuclear Elements (LINEs), LTRs, DNA transposons and non-*Alu *Short Interspersed Nuclear Elements (SINEs) (see Materials and methods for details). For each of these sets, we calculated the ERA distributions for the same SRE sets as before. As can be seen in Figure 7e, all the pseudo-exon sets show absolute ERA values close to zero. Moreover, they do not present the ERA pattern expected to favor exonization. Indeed, pseudo-exons overlapping DNA transposons and LINEs have negative ERA mean values for ESEcomb. The exception seems to be for LTR pseudo-exons, which have positive ERA values for ESEcomb and negative for ESScomb, but with very low absolute values. This suggests that the high rate of *Alu *exonization may simply be due to their lack of silencers.

Although *Alu *elements do not seem to have a strand bias inserting within introns in human genes, protein-coding exons are mostly created from anti-sense *Alu *elements [40]. In fact, we could only find 64 cases of sense *Alu *pseudo-exons. In comparison, we could find more than 30,000 *Alu *pseudo-exons with the *Alu *in anti-sense. This difference can be explained by the efficiency of the splice sites [22,23], as sense *Alu *exons do not contain the strong poly-pyrimidine tract typical of anti-sense ones. Furthermore, most PS exons overlapping anti-sense *Alu *elements are normally 80 bp long or greater. These lengths correspond, in most cases, with the most commonly used splice sites created by the anti-sense *Alu *[46] (data not shown). In order to understand the differences in exonization levels, we compared the properties of these two under-represented cases, sense *Alu *exons and anti-sense *Alu *exons shorter than 80 bp, making use of pseudo-exons overlapping these elements: pseudo-exons overlapping and *Alu *in the same orientation (pseudoSS-*Alu*) and pseudo-exons smaller than 80 bp that overlap an *Alu *in the opposite strand (pseudoSH-*Alu*) (see Materials and methods for details). Interestingly, both sets have a different content of splicing regulatory motifs with respect to anti-sense *Alu *pseudo-exons (pseudo-*Alu*) bigger than 80 bp (Figure A5 in Additional data file 1). Even though pseudoSS-*Alu *shows for both sets of ESEs higher exonic densities with respect to the adjacent intronic regions (Figure A5A and A5B in Additional data file 1), no differences are observed for ESSs (Figure A5C in Additional data file 1). This leads to positive ERA values for ESEs (0.091 and 0.086) but close to zero values for ESSs (-0.023). On the other hand, pseudoSH-*Alu *shows negative ERA values for ESEs (-0.167 and -0.168) and close to zero mean ERA values (-0.040) for ESSs (Figure A5D in Additional data file 1). Thus, both pseudoSS-*Alu *and pseudoSH-*Alu *exons have ERA values for ESSs close to zero, as opposed to anti-sense *Alu *pseudo-exons and PS exons overlapping *Alu*s, which have very large negative ERA values for ESSs. This suggests that the higher content ESSs make sense *Alu*s and regions smaller than 80 bp within anti-sense *Alu*s less prone to exonization.

## Discussion

We have analyzed the regulatory requirements for exonization and how splicing regulation changes throughout the exon lifespan by comparing the splicing regulatory properties of human internal protein-coding exons classified into three age groups: primate specific (PS), mammalian specific (MS) and vertebrate and older exons (VO). Most of the PS exons are alternatively spliced and show low inclusion levels. We find only about 5% of PS exons to be constitutive, whereas previous analyses [1] report about 60% of exons to be constitutive in a PS set. This difference can be explained by the fact that our method is more stringent; hence it is less likely that older exons are misclassified as PS ones; and could also be due to the fact that we discarded exons that may have originated from tandem duplication events, which are copies of pre-existing exons and would be similar to older ones. Furthermore, we find that PS exons are more likely to maintain the reading frame, indicating an additional pressure to reduce their impact in protein-coding regions. This increased frequency of symmetric exons observed in the PS set, especially in highly included exons, is likely to be related to the fact that the isoform including the exon is a novel one. On the contrary, for MS and VO, lowly included exons are more frequently symmetric. This suggests that in these cases, or in a significant fraction of them, the ancestral form might have been constitutively spliced, having more recently become alternative. This provides extra evidence supporting the hypothesis that the appearance of novel isoforms is favored when their impact is reduced. In this scenario AS acts as a key player allowing the incorporation of novel regions in mature transcripts and resulting products, establishing a close relationship with the process of exon creation [3].

We have also investigated the splicing regulatory requirements for *de novo *exonization. We observed that real exons have significantly different content of regulatory elements compared with pseudo-exons. However, there are also significant differences in the flanking introns. Indeed, we observe significant differences in the adjacent intronic content of SREs that were originally classified as exonic. Intronic regions adjacent to real splice junctions present lower densities of ESEs and higher densities of ESSs when compared to regions adjacent to pseudo-exons. This does not necessarily imply that such motifs are active in these regions. However, these differences could be the result of a balance with other nearby regulatory elements.

As exonization is related to changes in the exonic and in the adjacent intronic regions, they should both be taken into account. Accordingly, we defined a single measure, ERA, which encapsulates the regulatory content of each exon and its flanking introns. We have shown that this measure can differentiate better real exons from pseudo-exons than the exonic or intronic densities alone. For the three motif sets used, ERA provides the best discriminatory power. We also found that ESEcomb and ESScomb, which are combined sets of ESEs and ESSs, respectively, performed better than the individual sets alone. Another result worth mentioning is the fact that these two computational defined sets, performed better than the experimentally determined SRall set. The fact that these two sets have been partly defined based on exon versus intron and exon versus pseudo-exon comparisons might favor their discriminative power when using exonic density as a factor. Interestingly, the same holds true for intronic density at a lower extent. Relative to a third set of SR protein binding sites (SRall), we observed that SF2/ASF binding motifs perform consistently better than SC35, SRp40 and SRp55 binding sites. We thus expect that ERA or any other measure that takes into account local differences in motif content will contribute to the improvement of current methods of splice site and exon prediction.

We observed that the difference in inclusion levels between the different exon age groups cannot be fully attributed to the splice site strength. Further studies on regulatory content have shown that PS exons have smaller differences in ESE motifs between exons and flanking introns than conserved exons, that is, they are more similar to pseudo-exons than to older exons. This indicates that a minimal amount of regulatory motifs is needed for exonization. Moreover, the greater difference in the local density of regulators for older exons means that they have acquired a consolidated set of regulators. In fact, our results indicate that the relative density of regulatory motifs increases with time, and at a higher rate in MS exons compared to VO exons. Additionally, we found that exons become more established, that is, exhibit higher inclusion, by acquiring more enhancers relative to the flanking introns and by increasing the density of silencers in introns relative to the exons they flank. This is ultimately reflected in the higher ERA absolute values obtained.

Our analyses suggest that the local sequence context in which the exon is located plays a role in how splicing is regulated. Although there is no direct experimental evidence of a mechanism in which the spliceosome senses the local densities of splicing motifs, there is plenty of evidence of how the relative abundance of motifs can determine the splicing phenotype. It has been shown previously that the density of motifs close to a splice site affects the splicing outcome [31]. In particular, exonic regions that were intronized due to mutations to splice-sites have less ESEs and more ESSs than average exons, and that intronic regions that were exonized upon creation of cryptic splice-sites in introns had more ESEs and less ESSs than normal introns [47]. This establishes a gradient of densities between the different regions classified according to splicing phenotype, similar to the one we find here. There is also evidence that some splicing regulatory motifs in exons and introns function in clusters [48-50], and that multiple ESEs increase additively the efficiency of splicing [51,52]. Since we observe that ESEs and ESSs can occur by chance almost anywhere in exons and introns [29,31], a local compensation in the density of motifs seems to be necessary to maintain a specific regulation [53], and this is reflected in the local differences between exons and introns, which we can measure using ERA.

Finally, we have also investigated the role of splicing regulatory elements in the exonization of TEs, which may account for 42.9% of PS exons. When untranslated regions (UTRs) are considered, the proportion of PS exons overlapping with TEs is higher [1]. In fact, it has been recently reported that exonization of TEs occurs more abundantly in UTRs [40]. Thus, new exons originating from TEs are accepted in protein-coding regions at a much lower rate than in UTRs. On the other hand, most of the new exons overlapping TEs have been found to introduce in-frame stop codons [40]. Many exonizations of TEs may occur as errors of the splicing mechanism, and are, therefore, less frequently included in the protein and, subsequently, are more often tolerated in UTRs. Since we started from a set of protein-coding exons, our PS exons are already part of an open reading frame, and can be considered as recently established, that is, have become accepted into the protein-coding region at low inclusion rates.

We observed that in most of the cases the *Alu *element overlaps the PS exon on the anti-sense strand, and that these are characterized by having a striking lack of silencers compared to the surrounding introns. As introns can be considered as regions with a basal density of splicing silencers [27,29], the insertion of an anti-sense *Alu *therefore creates a local desert of splicing silencers in the intronic region into which they are inserted. Thus, the frequently observed *Alu *exonization might not only stem from the presence of optimal splice sites, but also from the creation of an environment favorable for exonization. Interestingly, *Alu *pseudo-exons with overlap on the sense strand and those in anti-sense shorter than 80 bp have over-representation of ESSs in the exonic region, providing a possible explanation as to why they are not so frequently exonized.

In the human genome there are around one million Alu copies, 66% of which accumulate in intronic regions[40]. We found approximately 256,000 *Alu *pseudo-exons with splice sites scoring above the first quartile of the distribution of scores for real splice sites, which fall within an intron flanked by protein-coding exons, and for which there is no evidence of exonization from ESTs, cDNAs or proteins. From these pseudo-exons, 15,048 (5.9%) are bigger than 80 bp, have a length multiple of three and have no stop-codons in frame. Moreover, 6795 (45,1%) of these are conserved in chimp and macaque with conserved flanking AG and GT dinucleotides. One possible reason why these conserved *Alu *pseudo-exons do not appear to be included in the mature transcript is because they have not been detected yet in EST/cDNA sequencing experiments. However, considering the extensive EST evidence that is available for human, one can assume that most of these pseudo-exons are, in fact, silenced or are not recognized by the spliceosome. After analyzing the regulatory content of these candidates, we observed that the ERA values differ strikingly from the *Alu *exons in all sets of SREs, suggesting that insufficient difference in density of SREs between the potential exon and corresponding flanking introns prevent their exonization (Table A4 in Additional data file 1). This provides further support to the idea that a minimum regulatory content is required for *de novo *exonization.

## Conclusions

Our results suggest that specific sequence environments might be required for exonization. Namely, regions with lower ESS content contrasting with the surroundings may be more prone to exonization. Also, exon creation may require the acquisition of a sufficient number of ESEs. All this supports the notion that *de novo *exonization is more likely to occur when there is a sufficient difference in the density of splicing regulatory elements on either side of optimal splice sites. This, in fact, suggests a mechanism of exon creation and establishment in human. New exons appear with low inclusion level, as they do not have a sufficient amount of ESEs. In this context, *Alu *elements play a crucial role in *de novo *exon creation in primates. With time, the establishment of an exon is determined by the accumulation of ESEs. In parallel, the lack of ESSs plays an important role in distinguishing an exon from the adjacent introns. This acquisition of regulatory elements along with the differentiation with respect to the intronic context determine the establishment of an exon in the mature transcript.

In summary, exon establishment is determined by the acquisition of splicing regulation at a local level and, as shown, this can be measured using a specifically devised measure, the ERA. This measure can, in fact, distinguish better real exons from pseudo-exons than exonic or intronic densities of splicing motifs alone. We therefore conclude that local differences in motif densities affect splicing decisions and, subsequently, the recognition of exons. We expect that measures that take these differences into account will provide an improvement on standard exon and gene prediction methods.

## Materials and methods

### Datasets

Gene annotations for *Homo sapiens *(NCBI36, Apr 2006), *Mus musculus *(NCBI m36, Apr 2006), *Bos taurus *(Btau 2.0, Dec 2005), *Gallus gallus *(WASHUC 1, Dec 2005) and *Tetraodon nigroviridis *(TETRAODON 7, Sep 2004), and orthologous gene pairs between these species were downloaded from Ensembl [54]. From the set of orthologs, only unique best reciprocal hits were kept. Genes that had ambiguous orthologous assignations, that is, linked to more than one potential orthologous sequence in the other genome, were eliminated. EST, mRNA and RepeatMasker mappings were retrieved from UCSC Genome Browser Database [55].

### Alignment of exon-intron structures

Transcripts and coding sequences for each gene were projected onto the genomic sequence producing an array-like structure of genomic regions. These structures were then aligned between pairs of orthologous genes using information about the splice sites and exon phases. Orthologous genes from closely related species generally have high conservation of their exonic structure. Taking this into account, we performed comparisons between all splice sites from one gene against all those from its orthologue. A score was defined using the sequence identity between 40 nucleotides around the splice junctions (20 nucleotides upstream and 20 nucleotides downstream of each splice site) and the exon phase. All these scores were placed in a matrix, where every entry represents the score from the comparison of two splice sites from the orthologous gene pair. Subsequently, using a dynamic programming algorithm with this matrix, we identified the putative orthologous splice sites. This was done pair-wise between all five species. From this calculation we could detect orthologous exons and exons with potentially no orthologue in another genome.

### Classification of exons according to evolutionary ages

We considered those exons with the following properties: internal, protein-coding, longer than 30 nucleotides and without 3' or 5' AS. This last condition was required to guarantee that both regions upstream and downstream of the exon are fully intronic. The flanking introns were also required to be longer than 30 nucleotides each. Additionally, only exons with canonical splice sites (AG/GT) were considered. These requirements were necessary for the correct analysis of the densities of regulatory sequences (see below). In order to obtain the exons belonging to the three different age classes, comparisons using three species were performed. If a particular exon was present in one species (reference species) and absent in the most closely related one (target), this could mean that either that exon was created in the reference species or that it was lost in the target one. To resolve this question a third species (more distantly related to the reference) was used as out-group, to infer if the exon was present in the common ancestor of the first two species. Three different age classes were defined: primate specific (PS), mammalian specific (MS) and vertebrate and older (VO). PS exons were defined as human exons that were not present in mouse or cow (strictly speaking, PS exons are human exons that are possibly also present in other primates). MS exons were defined as human exons conserved in mouse and cow, but not present in chicken or Tetraodon. Finally, VO exons were defined as human exons that are conserved in all the other four species. Exons that were aligned to orthologous exons were considered as conserved. Exons that did not have an alignment and were located between, but not necessarily adjacent to, conserved exons were considered to be candidates for PS or MS exons. These candidates were then compared with TBLASTN against the region in the orthologous genes spanned between the nearest alignable splice sites. If any significant result was produced (e-value < 0.0005), that exon was discarded. In this way we do not consider as non-conserved exons that are evolving at a faster rate. In order to reduce the possibility that the remaining exons could have been originated by segmental duplication, exons that showed more than 80% similarity over 40% of coverage with respect to other exons from the same gene were discarded. As a final filter, we only kept exons that were supported by EST or mRNA evidence. As the search uses very stringent criteria of sequence conservation, we do not expect the sizes of the obtained age groups to necessarily reflect the real number of exons belonging to these age categories in the human genome.

### Pseudo-exon sets

In addition to the three age groups, we built sets of pseudo-exons overlapping and not overlapping TEs. Pseudo-exons are defined as intronic sequences of length comparable to exons, flanked by canonical splice sites and not present in any ESTs or cDNAs. Moreover, these have have a length multiple of three and with no stop codons in frame. Using the RepeatMasker annotations retrieved from UCSC Genome Browser Database [55], repetitive and repetitive-free regions were determined from intronic regions located between protein-coding exons. As we needed to score splice sites and obtain pseudo-exons of size 30 nucleotides or longer, we considered regions bigger than 56 nucleotides (20 on the acceptor side + 30 exonic + 6 on the donor side). Then, all the candidate splice sites in the sense strand that score above the first quartile of all human protein-coding exons were taken and all the pairs of acceptor and donor producing an exon bigger than 30 nucleotides were determined. Finally, we also applied filters to extract exons with a length multiple of three and that did not produce a stop codon in frame. We obtained a set of pseudo-exons not overlapping any TE (pseudo-INT) and five sets of pseudo-exons overlapping the four main classes of repeats (SINEs: pseudo-MIR and pseudo-*Alu*; LINEs: pseudo-LINE; DNA repeats: pseudo-DNA; and LTRs: pseudo-LTR).

*Alu *elements contain several possible 5' and 3' splice sites [22,23]. However, not all are commonly used. The splice sites most generally used in exonized anti-sense *Alu*s make up for exons of a size of around 80 bp and bigger [46]. From our PS set, 95% of exons overlapping *Alu*s are of length 80 bp or longer. Accordingly, all pseudo-exons analyzed were taken to be 80 bp or longer. We also created two additional sets of pseudo-exons following the above defined criteria: pseudo-exons overlapping sense *Alu *elements (pseudoSS-*Alu*) and a set of short (smaller than 80 nucleotides) pseudo-exons overlapping anti-sense *Alu *elements (pseudoSH-*Alu*).

### EST inclusion level

EST alignments were retrieved from UCSC Genome Browser Database [55] and compared with the Ensembl [54] annotations. For each exon, the percentage of EST inclusion level is defined as:

$$\text{\% inclusion}=100\frac{{\text{N}}_{\text{i}}}{{\text{N}}_{\text{i}}{\text{ + N}}_{\text{s}}}$$

where N_i _is the number of ESTs including the exon and N_s _the number of ESTs that cover the genomic region of the exon but skip it. Only exons with N_i _+ N_s _≥ 10 were considered. Some exons have zero EST inclusion, as all the corresponding ESTs show exon skipping, but their existence is supported by mRNA and/or protein evidence.

### Density of repetitive elements

RepeatMasker mappings overlapping exons and both upstream and downstream introns were retrieved from UCSC Genome Browser Database [55]. For the main four categories of elements (SINEs, LINEs, LTRs, DNA) we calculated the intronic densities as the fraction of cases where a particular type of element overlaps each base. Also, we tested whether exons belonging to different age groups overlapped any of these elements.

### Splice site strength

We scored all splice sites using position weight matrices for the human donors and acceptors. We considered positions (-20 nucleotides to +3 nucleotides) relative to the acceptor site and (-3 nucleotides to +6 nucleotides) to the donor site.

### Relative abundance of regulatory motifs

We used three sets of regulatory motifs: 666 ESE hexamers [42], which we call ESEcomb, built from the combination of 238 RESCUE-ESE hexamers [28] and 2,069 PESE octamers [29]; all possible words obtained from the four position-specific weight matrices for SR-protein binding sites from ESE-finder (SF2/ASF, SC35, Srp40 and Srp55) using the proposed thresholds [43], which we called SRall; and 386 ESS hexamers [42], which we called ESScomb and which were built from a combination of 176 FAS-ESS hexamers [27] and 974 PESS octamers [29]. Some of these sets were partially defined based on exon/intron and on exon/pseudo-exon enrichment [28,29]. Further, we introduced a new measure called the ERA. For each exon, and for a given set of motifs, we define the value r, calculated from the density of motifs in the exon (density_exon_) and surrounding intronic sequences (density_intron_) as follows:

$$r=\frac{{\text{density}}_{\text{exon}}-{\text{density}}_{\text{intron}}}{\max\;({\text{density}}_{\text{exon}}{\text{,density}}_{\text{intron}})}$$

where density_exon _and density_intron _are calculated as the fraction of positions covered by the motifs in an exonic and intronic sequence, respectively. To calculate exonic densities we considered the whole exon length and for the intronic densities we took 200 bp from adjacent intronic regions (100 on each side). The results did not differ when considering only the regions from both exon ends (Figure A6 in Additional data file 1). We did not take into account positions that are part of the splice site signals - namely, 3 exonic and 6 intronic for the donor site, and 3 exonic and 20 intronic for the acceptor site - as these are biased in sequence content. We considered only exons of at least 46 bp and with flanking introns of at least 126 bp. The analyses performed on the SRE sets were also performed on the individual sets from which they were built (see Additional data file 1 for further details).

### Classification of real versus pseudo-exons

We considered two initial groups consisting of 10,000 real exons and 10,000 pseudo-exons not overlapping any TE. These were merged into a single group for assessment of classification accuracy based on SRE content. Three sets of SREs were taken (ESEcomb, SRall and ESScomb) and three different measures (exonic density, intronic density and ERA) were tested as real/pseudo-exon classifiers. A 10-fold cross-validation was performed by randomly splitting the initial set into 10 parts of equal size. Each of these parts was scored using the remaining nine as training data for determining the cut-off leading to the highest accuracy. The performance was determined by calculating the accuracy value obtained in the test set. Additionally, in order to estimate the performance of each classifier, for all possible cut-off values, false positive rates and true positive rates were determined for each subset and ROC curves and AUCs were calculated.

### SRE functional conservation score

For a given alignment of a human/mouse orthologous exon pair and a given SRE set, we calculate the FCS as defined in [11], that is, FCS = N/M, where N is the number of positions in the alignment that are covered by motifs in both species and M is the number of positions in the alignment that are covered in either human, mouse or both. FCS varies between 0 and 1, where 1 means that all bases covered by motifs in human are also covered by motifs in mouse; and 0 that none of the bases covered by motifs in one species is covered in the other.

## Abbreviations

AS: alternative splicing; AUC: area under the curve; ERA: exonic relative abundance; ESE: exonic splicing enhancer; ESS: exonic splicing silencer; EST: expressed sequence tag; FCS: functional conservation score; FPR: false positive rate; LINE: long interspersed nuclear element; LTR: long terminal repeat; MS: mammalian specific; PS: primate specific; ROC: receiver operating characteristic; SINE: short interspersed nuclear element; SRE: splicing regulatory element; TE: transposable element; UTR: untranslated region; VO: vertebrate and older.

## Authors' contributions

AC and EE conceived the project and wrote the manuscript. AC carried out the analyses. All authors read and approved the final manuscript.

## Additional data files

The following additional data are available with the online version of this paper. Additional data file 1 contains all additional figures (Figures A1-6), additional tables (Tables A1-4) and corresponding captions. Additional data file 2 contains two tab separated files with table listings of the exons and *Alu *pseudo-exons used.

## Supplementary Material

Additional data file 1 Additional figures (Figures A1-6), and additional tables (Tables A1-4) Click here for file

Additional data file 2 Exons and *Alu *pseudo-exons used. Click here for file
