# Supplementary material for: Exon creation and establishment in human genes
Source: Genome Biol. 2008 Sep 23;9(9):R141. doi: 10.1186/gb-2008-9-9-r141 (PMC2592719; doi:10.1186/gb-2008-9-9-r141)
Supplement: Additional data file 1 — Additional figures (Figures A1-6), and additional tables (Tables A1-4) [file gb-2008-9-9-r141-S1.pdf]

Additional File 1

Title:

Exon creation and establishment in human genes

Authors:

André Corvelo [1,3], Eduardo Eyras [1,2 §]

[1]Computational Genomics, Universitat Pompeu Fabra, Aiguader 88, Barcelona, 08003, Spain

[2]Catalan Institution for Research and Advanced Studies, Passeig Lluís Companys 23, Barcelona, 08010, Spain

[3]Graduate Program in Areas of Basic and Applied Biology, Universidade do Porto, Praça Gomes Teixeira, Porto, 4099-002, Portugal

[§]Corresponding author

Email addresses:

AC: acorvelo@imim.es

EE: eeyras@imim.es

Additional Figures

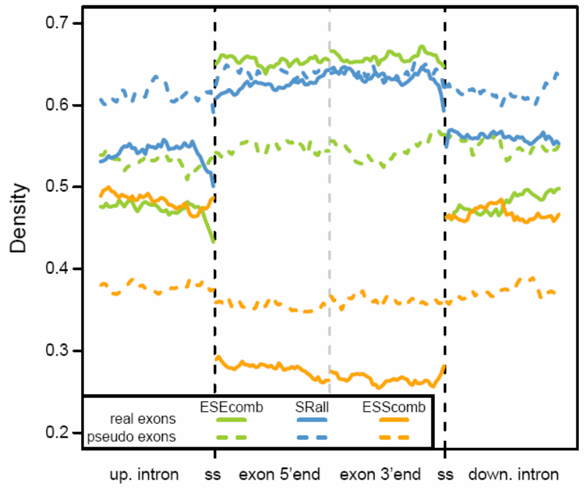

Figure A1 – SRE positional frequency for real and pseudo exons.

Positional frequency (y-axis) for each position (x-axis) of each motif set is given as the proportion of cases in which that position appears covered by elements of the motif set. The regions corresponding to the splice sites, 20+3 at the acceptor sites and 3+6 at the donor sites, were not considered. After removing these positions, the frequency was calculated for the first and last 50 bases of the exon, and the 50 bases upstream the acceptor and downstream the donor in flanking introns.

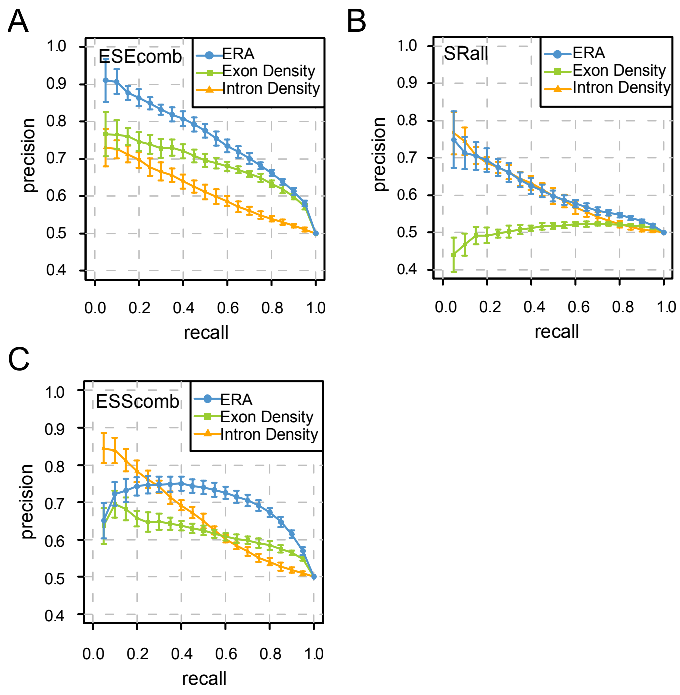

Figure A2 – Performance comparison in real / pseudo exon discrimination between different measures.

Precision-recall curves (vertically averaged) for exonic density, intronic density and ERA, using (A) ESEcomb, (B) SRall and (C) ESScomb as informative features. The average was calculated from ten splits of the data (see text for details).

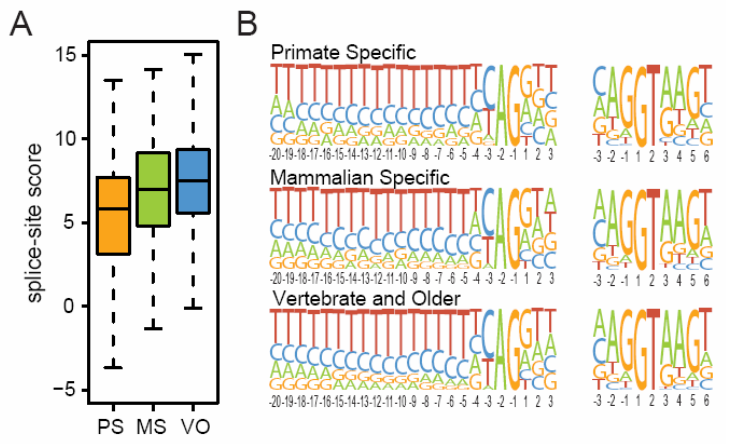

Figure A3 – Splice site scores and consensus for the three age groups.

(A) Combined donor and acceptor score distributions for the three age groups. The score is defined the sum of both donor and acceptor scores. (B) Logos for the acceptor (left) and donor (right) signals for the three age groups.

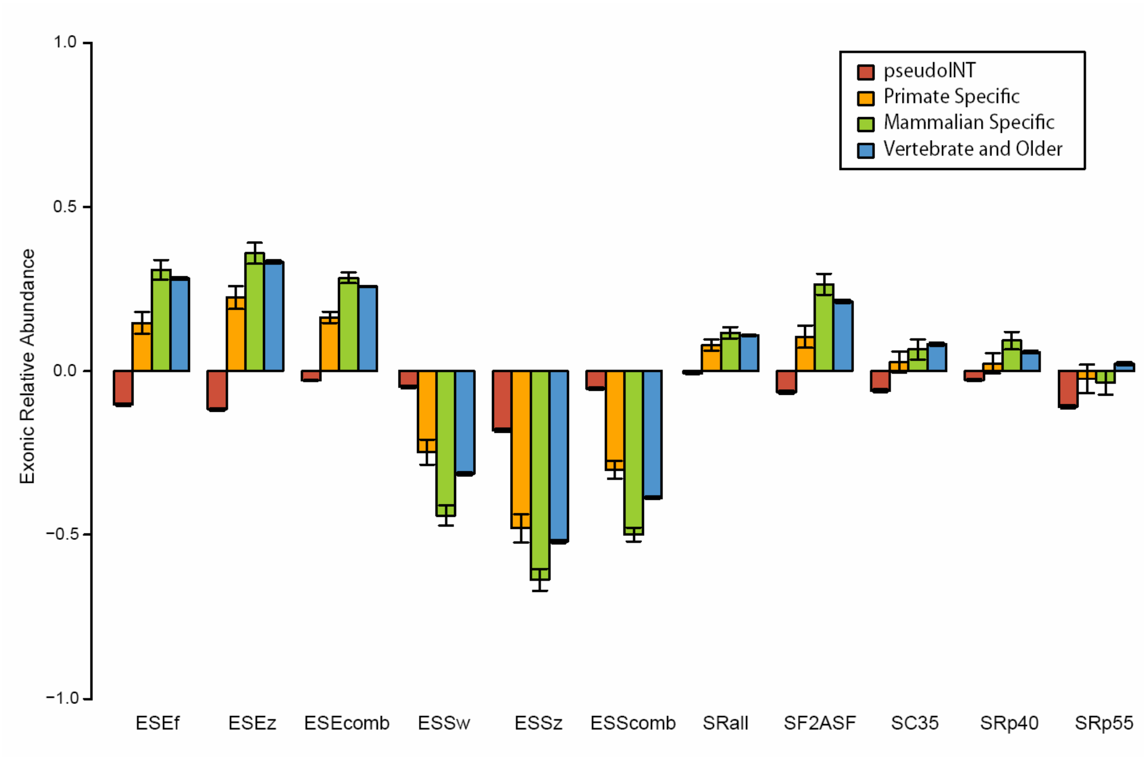

Figure A4 – SRE exonic relative abundance for the three age groups and an additional set of pseudo-exons.

Mean exonic relative abundance values for the three age groups (PS, MS and VO) and a set of pseudo exons not overlapping any repeats calculated for eleven SRE sets (ESEf, RESCUE-ESE [28]; ESEz, PESE [29]; ESEcomb, RESCUE-ESE + PESE [42]; ESSw, FAS-ESS [27]; ESSz, PESS [29]; ESScomb, FAS-ESS + PESS [42]; SF2/ASF [43]; SC35 [43]; SRp40 [43]; SRp55 [43]; Srall, SF2/ASF + SC35 + SRp40 + SRp55). Exons overlapping *Alu*´s were excluded from the PS set.

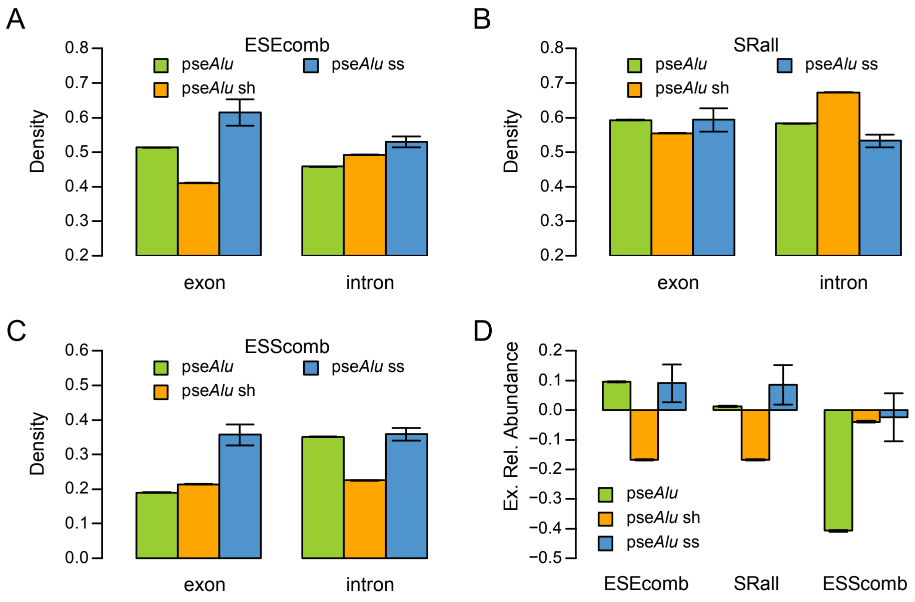

Figure A5 – *Alu* pseudo-exon SRE content.

Exonic and intronic densities of ESEcomb (A), SRall (B) and ESScomb (C) motifs on *Alu* pseudo-exons (pse*Alu*), short *Alu* pseudo-exons (pse*Alu* sh) and pseudo-exons derived from *Alu* elements inserted in the same strand as the gene (pse*Alu* ss). (D) Exonic relative abundance of ESEcomb, SRall and ESScomb motifs for the above mentioned pseudo-exon sets.

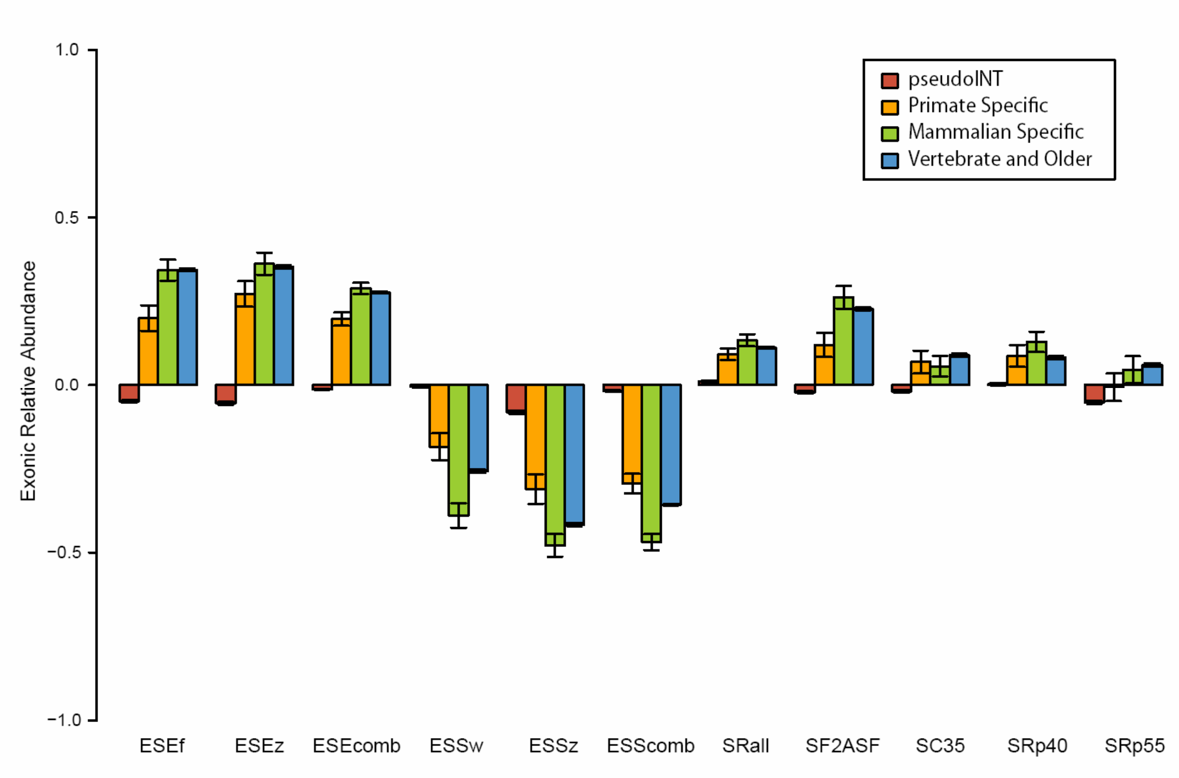

Figure A6 – SRE exonic relative abundance for the three age groups and an additional set of pseudo-exons using fixed length regions of 40nt upstream and downstream the splice sites.

Mean exonic relative abundance values for the three age groups (PS, MS and VO) and a set of pseudo exons not overlapping any repeats calculated for eleven SRE sets (ESEf, ESEz, ESEcomb, ESSw, ESSz, ESScomb, SRall, SF2/ASF, SC35, SRp40 and SRp55). Exons overlapping *Alu*'s were excluded from the PS set. SRE set abbreviations like in Figure A4.

Additional Tables

Table A1 – Different types of overlap with repetitive elements

For each age group is given the number and corresponding percentage of each kind of overlap observed.

Exon In – The exon is fully contained inside repetitive element; Acceptor Overlap – Only acceptor splice-site is overlapped by repetitive element; Donor Overlap – Only donor splice-site is overlapped; Both – Both donor and acceptor splice-sites are overlapped, but by different repetitive elements.

	Exon In		Acceptor Overlap		Donor Overlap		Both		
Exon set	N	%	N	%	N	%	N	%	Total
PS	105	68,18%	21	13,64%	20	12,99%	8	5,19%	154
MS	-	-	-	-	-	-	-	-	0
VO	1	3,45%	10	34,48%	18	62,07	-	-	29

Table A2 – Mean thresholds and accuracy for pseudo/real exon classification (10-fold cross-validation) using 11 different SRE sets. SRE set abbreviations like in Figure A4.

		Threshold		Accuracy	
SRE set	measure	Mean	St. Dev	Mean	St. Dev
ESEf	Exonic Density	0,223 a	0,000	0,671	0,010
	Intronic Density	0,300 b	0,011	0,533	0,012
	Exonic Relative Abundance	0,107 a	0,008	0,667	0,015
ESEz	Exonic Density	0,191 a	0,000	0,640	0,006
	Intronic Density	0,147 b	0,009	0,569	0,010
	Exonic Relative Abundance	0,160 a	0,000	0,663	0,009
ESEcomb	Exonic Density	0,564 a	0,000	0,672	0,008
	Intronic Density	0,450 b	0,010	0,588	0,010
	Exonic Relative Abundance	0,136 a	0,014	0,699	0,009
SF2/ASF	Exonic Density	0,168 a	0,000	0,549	0,007
	Intronic Density	0,130 b	0,008	0,587	0,012
	Exonic Relative Abundance	0,238 a	0,046	0,596	0,010

SC35	Exonic Density	0,191 a	0,000	0,538	0,005
	Intronic Density	0,200 b	0,000	0,561	0,011
	Exonic Relative Abundance	0,149 a	0,000	0,559	0,006
SRp40	Exonic Density	0,168 a	0,000	0,525	0,008
	Intronic Density	0,230 b	0,000	0,571	0,009
	Exonic Relative Abundance	0,039 a	0,044	0,534	0,009
SRp55	Exonic Density	0,034 a	0,001	0,553	0,008
	Intronic Density	0,176 b	0,006	0,510	0,010
	Exonic Relative Abundance	-0,836 a	0,000	0,551	0,008
SRall	Exonic Density	0,486 a	0,003	0,535	0,006
	Intronic Density	0,481 b	0,002	0,585	0,010
	Exonic Relative Abundance	0,163 a	0,019	0,580	0,012
ESSw	Exonic Density	0,152 b	0,018	0,612	0,008
	Intronic Density	0,170 a	0,000	0,547	0,018
	Exonic Relative Abundance	-0,067 b	0,013	0,619	0,012
ESSz	Exonic Density	0,166 b	0,000	0,603	0,005
	Intronic Density	0,243 a	0,008	0,601	0,011
	Exonic Relative Abundance	-0,318 b	0,023	0,646	0,008
ESScomb	Exonic Density	0,358 b	0,017	0,613	0,013
	Intronic Density	0,492 a	0,005	0,614	0,009
	Exonic Relative Abundance	-0,216 b	0,007	0,707	0,010

[a] minimum score cut-off for predicted real exons. [b] maximum score cut-off for predicted real exons.

Table A3 – ESR densities and Exonic Relative Abundance by age groups.

Mean (top), median (middle) and standard error (bottom) are given for every measure (Ed – exonic density; Id – intronic density; and ERA – Exonic Relative Abundance) using different SRE sets.

PS exons overlapping *Alu* elements were removed due to sequence bias (see manuscript). Additionally, a set of pseudo-exons not overlapping any repetitive element (pseudoINT) was included for analysis.

		ESEcomb			SRall			ESScomb		
Exon set	N	Ed	Id	ERA	Ed	Id	ERA	Ed	Id	ERA
pseudoINT	18839	0,504	0,510	-0,029	0,580	0,563	-0,006	0,373	0,387	-0,055
		0,518	0,515	-0,005	0,569	0,565	-0,018	0,357	0,385	-0,035
		0,001	0,001	0,002	0,001	0,001	0,002	0,001	0,001	-0,003
PS	213	0,626	0,506	0,164	0,639	0,571	0,080	0,247	0,383	-0,302

(non *Alu*)		0,638	0,515	0,177	0,660	0,585	0,110	0,240	0,360	-0,341
		0,009	0,009	0,017	0,010	0,009	0,017	0,010	0,010	0,027
MS	244	0,665	0,458	0,284	0,621	0,533	0,117	0,213	0,454	-0,499
		0,679	0,472	0,316	0,629	0,525	0,136	0,194	0,475	-0,563
		0,009	0,008	0,016	0,010	0,010	0,017	0,008	0,011	0,021
VO	11447	0,633	0,458	0,258	0,588	0,514	0,109	0,272	0,467	-0,387
		0,640	0,460	0,278	0,596	0,505	0,125	0,265	0,480	-0,418
		0,001	0,001	0,002	0,001	0,001	0,002	0,001	0,002	0,003

Table A4 – ESR densities and Exonic Relative Abundance for PS *Alu* exons and *Alu* pseudo-exons conserved in Primates.

Mean (top), median (middle) and standard error (bottom) are given for every measure (Ed – exonic density; Id – intronic density; and ERA – Exonic Relative Abundance) using different SRE sets.

Mann-Whitney *p*-values are also shown for comparisons between pseudo-*Alu* and PS-*Alu* exon sets.

		ESEcomb			SRall			ESScomb		
Exon set	N	Ed	Id	ERA	Ed	Id	ERA	Ed	Id	ERA
pseudo-*Alu*	6795	0,516	0,459	0,098	0,597	0,581	0,022	0,192	0,355	-0,411
(conserved in primates)		0,516	0,460	0,109	0,600	0,590	0,018	0,189	0,340	-0,467
		0,001	0,001	0,003	0,001	0,001	0,003	0,001	0,001	0,004
PS – *Alu*	77	0,597	0,412	0,276	0,649	0,524	0,177	0,150	0,451	-0,625
		0,593	0,415	0,306	0,650	0,520	0,220	0,125	0,430	-0,697
		0,013	0,012	0,030	0,012	0,014	0,026	0,011	0,013	0,036
p-value		$3,31x10^{-11}$	$3,23x10^{-4}$	$1,37x10^{-9}$	$4,14x10^{-5}$	$4,76x10^{-5}$	$5,38x10^{-7}$	$8,50x10^{-5}$	$7,34x10^{-11}$	$8,24x10^{-10}$
